# Physiological Versus Directed Pushing During the Second Stage of Labor: A Non-Randomized Quasi-Experimental Study of Maternal Recovery and Clinical Outcomes

**DOI:** 10.3390/healthcare14193319

**Published:** 2026-10-06

**Authors:** Alya Mansour Alshamrani, Nouran Essam Katooa, Safaa Gaber Aly Salem, Enas Mohamed Lotfy, Hanan Elsayed Mohamed Elsayed, Ahmed Azzam, Hala Ahmed Thabet

**Affiliations:** 1 Obstetrics and Gynaecologic Nursing Department, Faculty of Nursing, King Abdulaziz University, Jeddah 21589, Saudi Arabia; alya.m.alshamrani@gmail.com; 2 Maternity and Child Health Department, Faculty of Nursing, King Abdulaziz University, Jeddah 21589, Saudi Arabia; nkuttouaha@kau.edu.sa; 3 Department of Maternity, Obstetrics and Gynecologic Nursing, College of Nursing, Princess Nourah bint Abdulrahman University, Riyadh 11671, Saudi Arabia; 4 Public Health Nursing Department, Faculty of Nursing, Northern Border University, Arar 91431, Saudi Arabia; enas.mahmoud@nbu.edu.sa; 5 Woman’s Health and Midwifery Nursing Department, Faculty of Nursing, Mansoura University, Mansoura 35516, Egypt; alemam2100@yahoo.com (H.E.M.E.); rawan@mans.edu.eg (H.A.T.); 6 Department of Microbiology and Immunology, Faculty of Pharmacy, Capital University, Cairo 11795, Egypt; ahmed.abdelkareem@pharm.capu.edu.eg

**Keywords:** physiological pushing, directed pushing, second stage of labor, maternal fatigue, perceived energy, postpartum recovery, maternal outcomes, neonatal outcomes

## Abstract

**Background:** Pushing technique may influence maternal effort, labor progression, and birth outcomes, yet evidence comparing physiological and directed pushing remains inconsistent, particularly for early maternal recovery. This study compared the two approaches in relation to postpartum fatigue and perceived energy, second-stage duration, and selected maternal and neonatal outcomes. **Methods:** This non-randomized quasi-experimental comparative study included 120 women assigned during two sequential labor-unit implementation periods to physiological pushing (*n* = 60) or directed pushing (*n* = 60). No individual random allocation sequence was used. The physiological-pushing intervention was provider-guided open-glottis, short-duration pushing with urge-led initiation, whereas directed pushing used coached closed-glottis Valsalva efforts. The primary outcome was fatigue at 24 h postpartum. Covariate-adjusted regression was used for 24 h fatigue and perceived energy, mixed-effects models for repeated scores, proportional-odds regression for categorized second-stage duration, and exact methods for binary outcomes. Propensity-score overlap weighting evaluated the robustness of the primary result. **Results:** At 24 h postpartum, no between-group difference in fatigue was detected after adjustment for baseline fatigue, age, body mass index, education, gravidity, gestational age, first-stage duration, and oxytocin use (physiological-minus-directed difference, 0.20 points; 95% CI, −2.05 to 2.45; *p* = 0.860). Adjusted perceived energy was also similar (difference, −0.79 points; 95% CI, −2.18 to 0.59; *p* = 0.260). Across both groups, fatigue decreased and perceived energy increased over time (both time effects *p* < 0.001), with no group × time interaction. The overlap-weighted analysis supported the primary finding (difference, 0.25 points; 95% CI, −1.79 to 2.29; *p* = 0.809). Physiological pushing was associated with lower odds of occupying a longer second-stage-duration category in the adjusted proportional-odds model (adjusted OR, 0.20; 95% CI, 0.09 to 0.45; *p* < 0.001; Holm-adjusted *p* = 0.0007). None of the six binary maternal or neonatal outcomes differed after Holm correction. **Conclusions:** Compared with directed pushing, physiological pushing was not associated with lower fatigue or higher perceived energy at 24 h postpartum. Directed pushing likewise showed no consistent advantage over physiological pushing across the evaluated maternal and neonatal outcomes. The observed association between physiological pushing and lower odds of a longer second-stage duration represents an exploratory secondary finding and should be interpreted cautiously in view of the non-randomized design and the possibility of residual confounding.

## 1. Introduction

The second stage of labor extends from complete cervical dilatation to birth and requires the coordinated action of uterine contractions, fetal descent, and maternal expulsive effort. Its management is clinically important because a prolonged second stage is associated with maternal exhaustion, operative vaginal birth, postpartum hemorrhage, perineal trauma, and adverse neonatal outcomes [1,2]. Contemporary guidance therefore supports individualized care that promotes physiological birth while maintaining maternal and fetal safety [1,2]. Within this framework, the way women are supported to push is a modifiable component of intrapartum care.

Physiological, or spontaneous, pushing enables women to respond to the natural urge to bear down, typically using shorter expulsive efforts and relatively unrestricted breathing. Directed, or Valsalva, pushing uses provider-guided breath-holding and forceful bearing down during contractions [3,4]. This approach is consistent with physiological second-stage care that supports the woman’s own urge to push without prescribing the timing or duration of pushing [5]. Some trials have reported shorter active pushing, less fatigue, or more favorable neonatal indicators with this approach [6,7,8]. However, systematic reviews have not demonstrated a consistent advantage for labor duration, mode of birth, perineal outcomes, or neonatal condition [3,4,9,10]. Heterogeneity in parity, analgesia, maternal position, timing of pushing, breathing instructions, and outcome definitions complicates comparison across studies [3,4].

Most comparative research has emphasized obstetric and neonatal endpoints, whereas maternal recovery has been assessed less consistently. Fatigue has been measured with different instruments and at different postpartum intervals, and perceived energy has received comparatively little attention [7,10]. Consequently, it remains uncertain whether any advantage of physiological pushing extends beyond labor progression to sustained early postpartum recovery. The primary objective of this study was to compare fatigue at 24 h postpartum between physiological and directed pushing. Secondary objectives were to compare perceived energy at 24 h, fatigue and energy trajectories from the end of the first stage through 24 h postpartum, categorized second-stage duration, and selected maternal and neonatal outcomes.

## 2. Methods

### 2.1. Study Design and Setting

A non-randomized, two-group quasi-experimental comparative study was conducted in the delivery room and postpartum unit of the Maternity and Children Hospital in Makkah, Saudi Arabia, from December 2020 through May 2021. Physiological (spontaneous) pushing was compared with directed (Valsalva) pushing during the second stage of labor. The two protocols were implemented during sequential periods rather than concurrently. Reporting was informed by the Transparent Reporting of Evaluations with Nonrandomized Designs (TREND) statement [11], and intervention description was guided by the Template for Intervention Description and Replication (TIDieR) framework [12].

### 2.2. Participants and Group Allocation

The analytic cohort comprised 120 women with term, singleton, live fetuses in cephalic presentation who were receiving care for vaginal birth. Eligible consenting women were recruited consecutively during two sequential implementation periods until the prespecified target of 60 women per technique was reached. Physiological pushing was implemented from 1 December 2020 to 28 February 2021, followed by directed pushing from 1 March to 31 May 2021. Allocation was determined by the protocol in use when each woman entered the second stage of labor; no individual random sequence was generated. Equal group sizes reflected the prespecified 1:1 recruitment target rather than randomization or statistical matching. Women were eligible if they were 20–40 years of age, had a gestational age of 37–41 completed weeks, and met the predefined obstetric criteria for participation in vaginal birth. Women with multiple gestation, fetal malpresentation, planned cesarean delivery, or maternal medical or obstetric conditions requiring specialized obstetric management were excluded.

### 2.3. Pushing Interventions

Women in the physiological-pushing group were encouraged to begin bearing down in response to the spontaneous urge, maintain an open glottis with relatively unrestricted breathing, and use a comfortable position. Approximately five to six short efforts lasting 3–10 s were encouraged during each contraction. Women in the directed-pushing group received the routine coached Valsalva technique: at the onset of a contraction, they were instructed to inhale deeply, hold their breath, and push forcefully, generally repeating the maneuver three times per contraction. Routine maternal and fetal surveillance was otherwise provided to both groups. Accordingly, this intervention is operationally described as provider-guided open-glottis, short-duration pushing with urge-led initiation rather than as entirely uncoached spontaneous pushing. Instructions for both approaches were provided by the attending delivery-room nursing staff.

### 2.4. Measures and Outcomes

The prespecified primary outcome was the fatigue-domain score of the Visual Analogue Scale for Fatigue (VAS-F) at 24 h postpartum. The 18-item VAS-F includes 13 fatigue items and five perceived-energy items; higher scores indicate greater fatigue or greater perceived energy within the corresponding domain [13]. An Arabic-translated version was administered. Both domains were assessed at the end of the first stage of labor, 1 h postpartum, and 24 h postpartum. Secondary outcomes were perceived energy at 24 h, repeated fatigue and energy trajectories, categorized second-stage duration (<10, 10–19, 20–29, or ≥30 min), spontaneous vaginal delivery, blood loss ≤ 500 mL, intact perineum, good neonatal condition at 1 and 5 min, and no neonatal intensive care unit admission. Maternal blood loss was estimated during routine clinical care and categorized as ≤500 mL or >500 mL. Neonatal condition was based on the recorded 1 and 5 min Apgar assessments and neonatal intensive care unit admission. Second-stage duration was defined as the interval from documented complete cervical dilatation (10 cm) to birth, irrespective of when active pushing began. It was recorded in the prespecified categories <10, 10–19, 20–29, and ≥30 min; exact continuous durations were not available. The ≥30 min category therefore represents the open-ended upper category of the archived data rather than a validated clinical threshold.

Participant and intrapartum variables summarized by group were age, body mass index, education, residence, occupation, gravidity, exercise during pregnancy, Kegel exercise, smoking, gestational age, first-stage duration, membrane condition, oxytocin augmentation, and oxygen use. The adjusted models included age, body mass index, education, gravidity (primigravida versus multigravida), gestational age, first-stage duration, and oxytocin use. The corresponding baseline fatigue or energy score was also included in each 24 h model.

### 2.5. Sample-Size Calculation

The sample-size calculation was based on the prespecified primary outcome, the VAS-F fatigue-domain score at 24 h postpartum. Lam and McDonald reported 24 h fatigue standard deviations of 18.3 and 15.9 points for spontaneous and directed pushing, respectively, corresponding to a pooled standard deviation of 17.09 points [14]. An absolute 10-point between-group difference was used as a planning threshold. With a two-sided α of 0.05, 80% power, and 1:1 allocation, an exact two-sample t-test required 47 women per group. Allowing approximately 20% for attrition or incomplete follow-up yielded 47/(1 − 0.20) = 58.75 women per group, which was rounded to 60 per group and a total recruitment target of 120.

### 2.6. Data Completeness and Reliability

Pushing-group assignment, all prespecified covariates, and fatigue and perceived-energy scores were complete for all 120 participants. One membrane-rupture-duration value was missing; this variable was not included in the adjusted models. Conditional variables, including smoking frequency and reason for neonatal transfer, contained expected structural missingness. Internal consistency of the fatigue and perceived-energy domains was estimated separately at each assessment using Cronbach’s alpha.

### 2.7. Statistical Analysis

Continuous variables are summarized as mean (standard deviation), and categorical variables as number (percentage). Between-group differences in participant and intrapartum characteristics were summarized with absolute standardized mean differences rather than significance tests. Values < 0.10, 0.10 to <0.20, 0.20 to <0.50, and ≥0.50 were interpreted as negligible, small, moderate, and large imbalance, respectively. The primary 24 h fatigue analysis used ordinary least-squares regression with HC3 heteroskedasticity-robust standard errors and adjustment for baseline fatigue, age, body mass index, education, gravidity, gestational age, first-stage duration, and oxytocin use. The corresponding 24 h perceived-energy model replaced baseline fatigue with baseline energy. Adjusted physiological-minus-directed differences and 95% confidence intervals were estimated from each fitted model.

Repeated fatigue and perceived-energy scores were analyzed in separate linear mixed-effects models with fixed effects for group, time, the group × time interaction, and the revised covariate set, together with a participant-specific random intercept. Estimated marginal means and physiological-minus-directed contrasts were calculated at each assessment; within-domain contrasts were adjusted by the Holm method. Categorized second-stage duration was analyzed using unadjusted and covariate-adjusted proportional-odds models. The group-specific proportional-odds assumption was evaluated by comparing the common-effect model with a model allowing the pushing-group effect to vary across category thresholds. Pearson chi-square and Wilcoxon–Mann–Whitney tests were used as sensitivity analyses. Binary maternal and neonatal outcomes were compared using Fisher exact tests. Multivariable logistic models were fitted only when the smaller outcome class contained at least 10 observations. A prespecified family of secondary outcomes was controlled using the Holm method.

Robustness of the primary result was assessed with propensity-score overlap weighting. Propensity scores were estimated from age, body mass index, education, gravidity, gestational age, first-stage duration, oxytocin use, and baseline fatigue, after which the primary fatigue model was refitted using overlap weights and HC3 robust standard errors. Adequacy of the weighting procedure was evaluated by examining the distribution and overlap of propensity scores between groups, calculating the effective sample size after weighting, and assessing covariate balance using absolute standardized mean differences before and after weighting. All tests were two-sided, with α = 0.05. Analyses were performed in R version 4.5.3 using packages including tableone (version 0.13.2), psych (2.6.5), sandwich (3.1.1), lmtest (0.9.40), lme4 (2.0.1), lmerTest (3.2.1), emmeans (2.0.2), and ordinal (2025.12.29).

## 3. Results

### 3.1. Participant Flow and Group Characteristics

**Figure 1:**
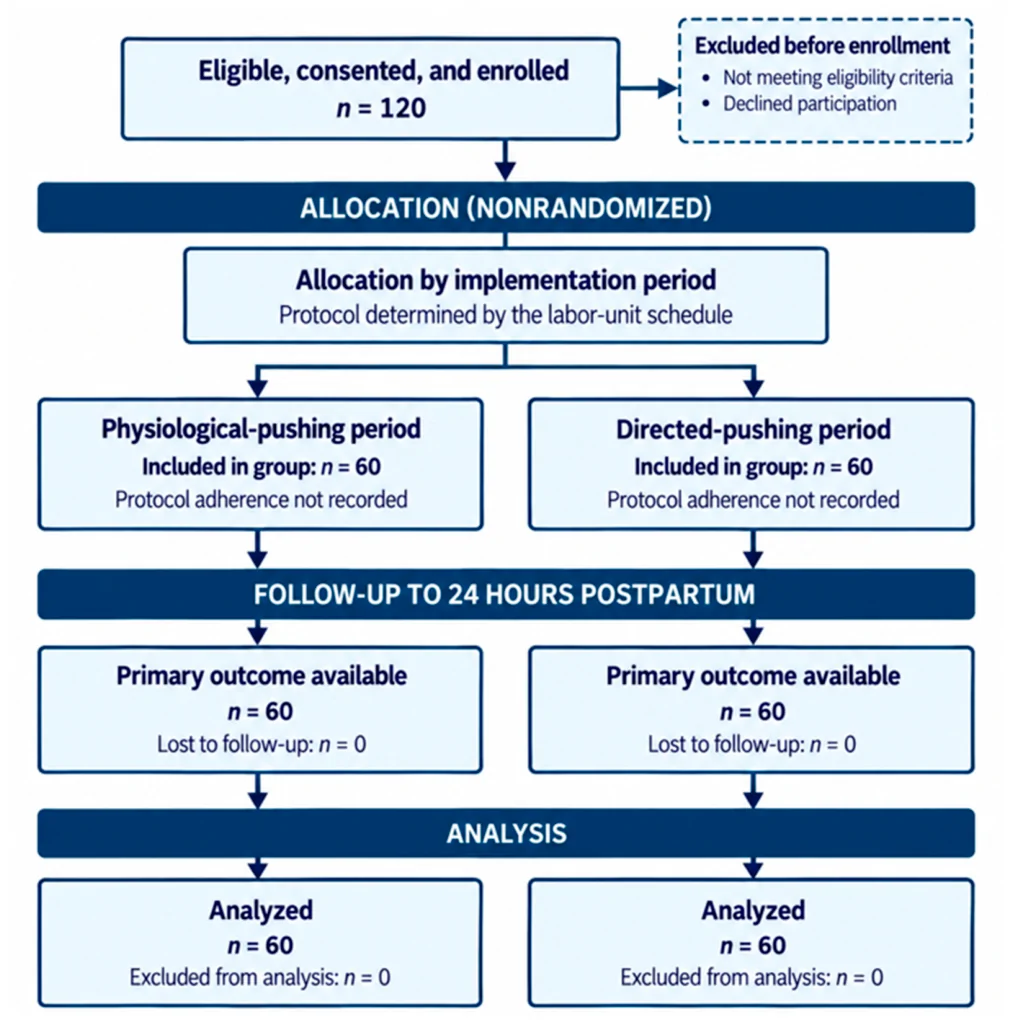
TREND-style participant flow showing enrollment, non-randomized allocation by sequential implementation period, 24 h follow-up, and analysis.

All 120 enrolled participants were retained in the analysis, with 60 women assigned during each implementation period and complete data for the primary outcome and adjustment variables (Figure 1). Counts of women assessed for eligibility, excluded before enrollment, or declining participation were not recorded in the archived study materials. Absolute standardized mean differences ranged from 0.036 to 0.488. Mean age was 25.97 years in the physiological group and 27.62 years in the directed group (SMD, 0.357), and mean body mass index was 27.65 and 30.03 kg/m^2^, respectively (SMD, 0.488). The SMDs were 0.438 for education, 0.409 for residence, 0.339 for gravidity, 0.332 for membrane condition, 0.320 for oxytocin augmentation, 0.266 for occupation, and 0.251 for first-stage duration (Table 1). Propensity-score distributions showed substantial overlap between the groups. After overlap weighting, the effective sample size was 96.3 of 120 participants, and covariate balance improved markedly, with the maximum absolute SMD decreasing from 0.493 before weighting to <0.001 after weighting (Figure S1 and Table S1).

### 3.2. Maternal Fatigue and Perceived Energy

Unadjusted scores at the three assessments are presented in Table 2. From the end of the first stage to 24 h postpartum, mean fatigue decreased from 46.40 to 31.25 with physiological pushing and from 47.13 to 30.92 with directed pushing. Over the same interval, mean perceived energy increased from 9.43 to 17.88 and from 9.60 to 18.52, respectively. Cronbach’s alpha values for fatigue were 0.504, 0.617, and 0.594 at the three assessments; the corresponding values for perceived energy were 0.735, 0.746, and 0.772.

### 3.3. Adjusted and Longitudinal Analyses

After adjustment for baseline fatigue, age, body mass index, education, gravidity, gestational age, first-stage duration, and oxytocin use, the physiological-minus-directed difference in fatigue at 24 h postpartum was 0.20 points (95% CI, −2.05 to 2.45; *p* = 0.860; Table 3). The adjusted physiological-minus-directed difference in perceived energy at 24 h was −0.79 points (95% CI, −2.18 to 0.59; *p* = 0.260).

For fatigue, the mixed-effects model yielded a time effect of F(2, 236) = 319.38 (*p* < 0.001), a group effect of F(1, 107) = 0.05 (*p* = 0.817), and a group × time interaction of F(2, 236) = 1.67 (*p* = 0.191). Holm-adjusted physiological-minus-directed contrasts were −0.97 (95% CI, −2.84 to 0.91; adjusted *p* = 0.623) at the end of the first stage, 1.30 (95% CI, −0.57 to 3.17; adjusted *p* = 0.519) at 1 h postpartum, and 0.10 (95% CI, −1.77 to 1.97; adjusted *p* = 0.916) at 24 h postpartum.

**Figure 2:**
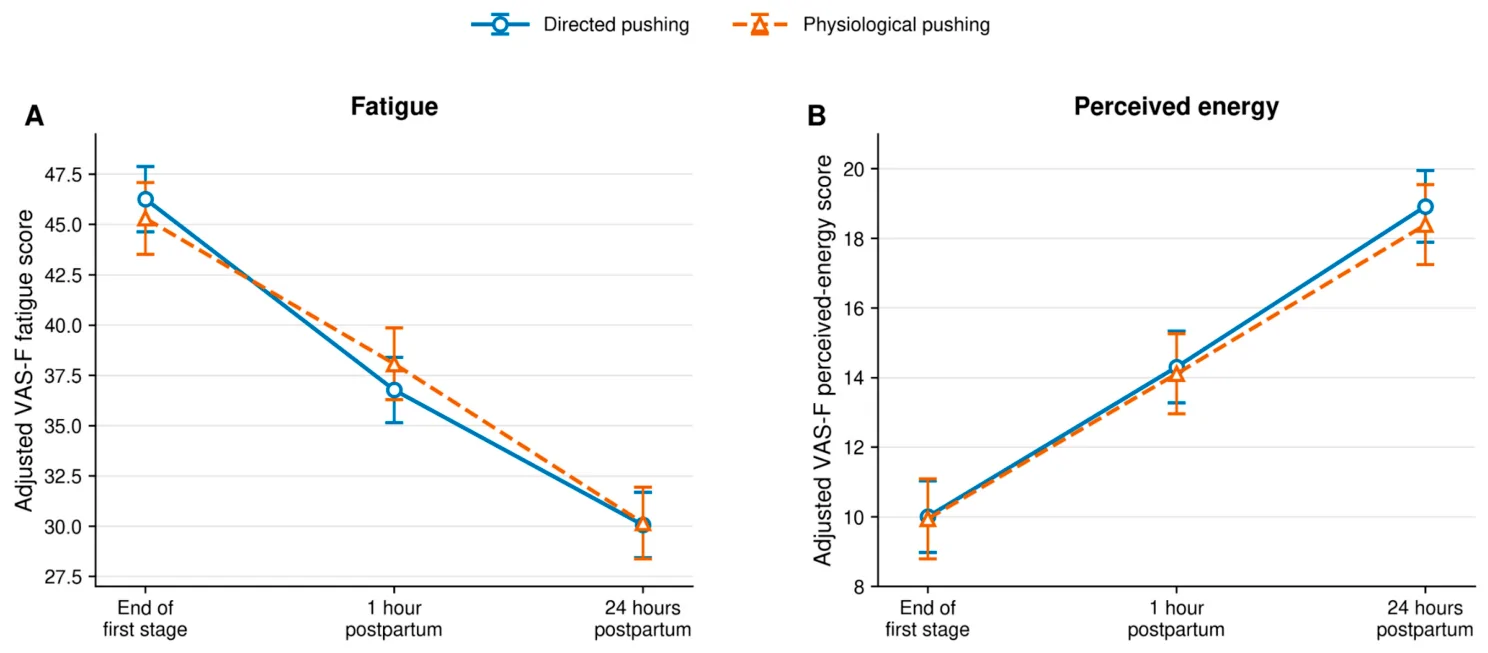
Covariate-adjusted estimated marginal mean VAS-F scores with 95% confidence intervals across the end of the first stage, 1 h postpartum, and 24 h postpartum: (**A**) fatigue and (**B**) perceived energy. Models adjusted for age, body mass index, education, gravidity, gestational age, first-stage duration, and oxytocin use. Circles with solid lines indicate directed pushing; triangles with dashed lines indicate physiological pushing.

For perceived energy, the mixed-effects model yielded a time effect of F(2, 236) = 315.52 (*p* < 0.001), a group effect of F(1, 107) = 0.39 (*p* = 0.534), and a group × time interaction of F(2, 236) = 0.24 (*p* = 0.785). The Holm-adjusted physiological-minus-directed contrasts had adjusted *p* = 1.000 at each time point. The overlap-weighted estimate for 24 h fatigue was 0.25 points (95% CI, −1.79 to 2.29; *p* = 0.809). After weighting, the maximum absolute standardized mean difference among measured covariates decreased from 0.493 to <0.001. Adjusted fatigue and perceived-energy trajectories are shown in Figure 2. Propensity scores ranged from 0.105 to 0.934 in the physiological-pushing group and from 0.104 to 0.793 in the directed-pushing group, with substantial distributional overlap (Supplementary Figure S1). The overall effective sample size after overlap weighting was 96.3 of 120 participants. Detailed covariate balance before and after weighting is provided in Supplementary Table S1; the maximum absolute SMD decreased from 0.493 before weighting to <0.001 after weighting.

### 3.4. Second-Stage Duration

**Figure 3:**
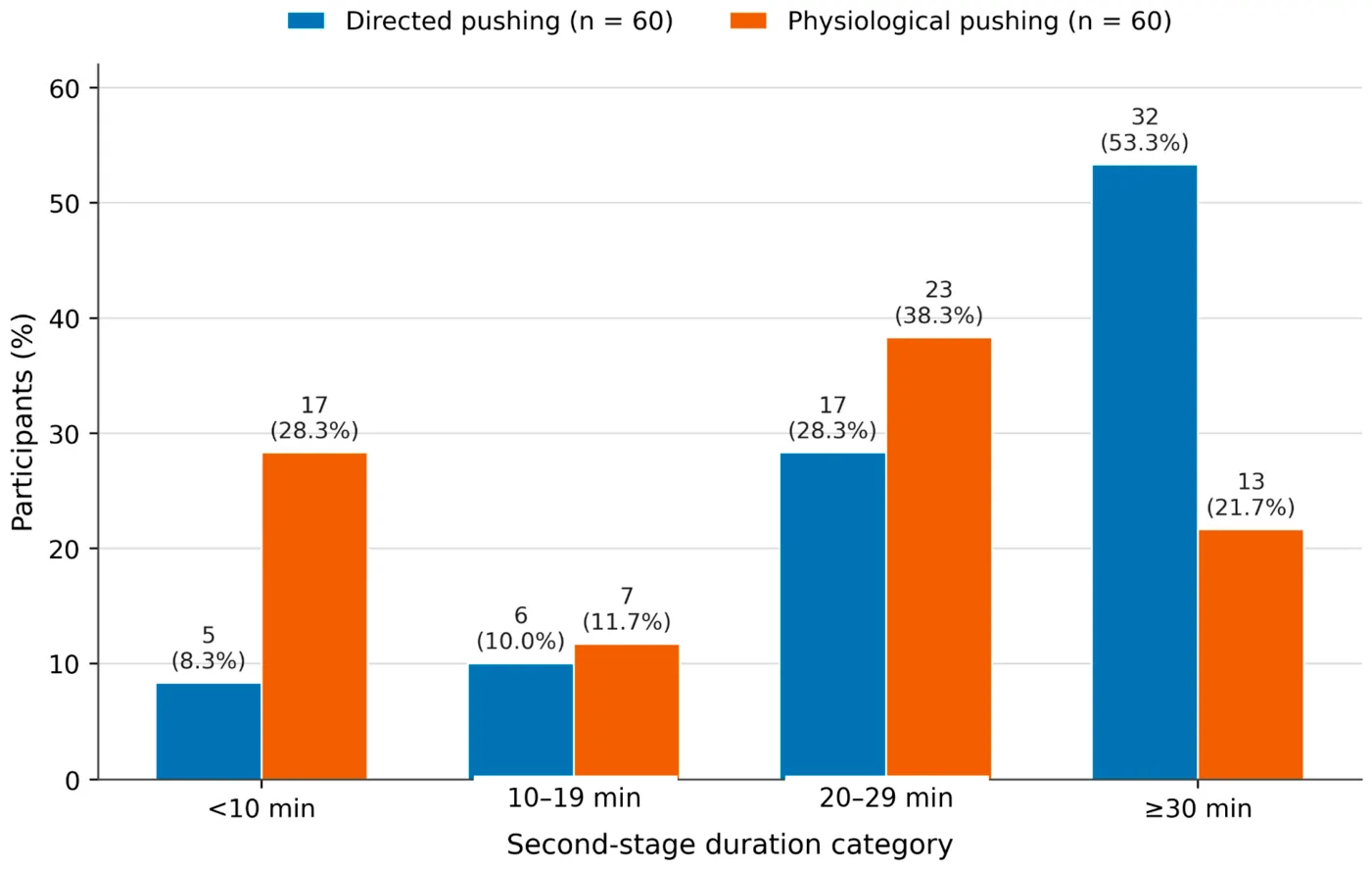
Distribution of second-stage duration categories by pushing group. Counts and percentages are shown above each bar; percentages sum to 100% within each group.

Seventeen women (28.3%) in the physiological group and five (8.3%) in the directed group delivered within <10 min; 13 women (21.7%) and 32 (53.3%), respectively, were in the ≥30 min category (Figure 3). The unadjusted proportional-odds model yielded an OR of 0.27 (95% CI, 0.13 to 0.53; *p* < 0.001). The adjusted proportional-odds model yielded an OR of 0.20 (95% CI, 0.09 to 0.45; *p* < 0.001; Holm-adjusted *p* = 0.0007). The likelihood-ratio *p* value for the group-specific proportional-odds test was 0.464. Sensitivity-analysis results were Pearson χ^2^ = 15.54 (df = 3, *p* = 0.0014) and Wilcoxon–Mann–Whitney *p* < 0.001.

### 3.5. Maternal and Neonatal Outcomes

Holm-adjusted *p* values for the six binary maternal and neonatal outcomes ranged from 0.401 to 1.000 (Table 4). Spontaneous vaginal delivery occurred in 55 women (91.7%) receiving physiological pushing and 60 (100.0%) receiving directed pushing (exact *p* = 0.057; Holm-adjusted *p* = 0.401). Intact perineum was recorded in 9 women (15.0%) and 17 (28.3%), respectively (exact *p* = 0.120; Holm-adjusted *p* = 0.719), and good neonatal condition at 1 min was recorded in 46 (76.7%) and 44 (73.3%) (exact *p* = 0.833; Holm-adjusted *p* = 1.000). Blood loss ≤500 mL and no neonatal intensive care unit admission were each recorded in 56 women (93.3%) in the physiological group and 59 (98.3%) in the directed group (exact *p* = 0.364; Holm-adjusted *p* = 1.000). Good neonatal condition at 5 min was recorded in 58 women (96.7%) in each group (exact and Holm-adjusted *p* = 1.000).

Adjusted logistic models were fitted for intact perineum and good neonatal condition at 1 min. The adjusted odds ratio comparing physiological with directed pushing was 0.69 (95% CI, 0.23 to 2.04; *p* = 0.499) for intact perineum and 1.90 (95% CI, 0.75 to 4.85; *p* = 0.179) for good neonatal condition at 1 min. Adjusted models were not fitted for the remaining outcomes because the smaller outcome class contained fewer than 10 observations.

## 4. Discussion

This study provides a recovery-centered comparison of physiological and directed pushing in a sequential-period, non-randomized clinical cohort. The equal group sizes of 60 women reflected the prespecified outcome-based recruitment target and 1:1 allocation ratio. Four principal findings frame the discussion. First, no between-group differences in fatigue or perceived energy were detected at 24 h after adjustment for the corresponding baseline scores and measured covariates. Second, both groups showed substantial recovery over time, with no differences in their fatigue or energy trajectories. Third, physiological pushing was associated with lower odds of being in a longer second-stage-duration category. Fourth, maternal and neonatal clinical outcomes showed no consistent between-group pattern after correction for multiple comparisons. Because the second-stage-duration association arose from a secondary outcome in a non-randomized study, it should be regarded as exploratory and remains susceptible to residual, temporal, and provider-related confounding. Overall, neither approach emerged as consistently superior across the evaluated outcomes. This pattern is broadly consistent with the Cochrane review and the recent meta-analysis by Kownaklai et al., which reported outcome-specific and sometimes inconsistent findings across labor, maternal, and neonatal outcomes [3,10].

The primary maternal-recovery analysis focused on fatigue at 24 h postpartum, an outcome less consistently evaluated than delivery mode or neonatal condition. The adjusted physiological-minus-directed difference was 0.20 points (95% CI, −2.05 to 2.45), and the overlap-weighted sensitivity analysis produced an estimate that was similarly close to zero. These results indicate that no between-group difference was detected at 24 h. However, they should not be interpreted as formal evidence of equivalence because no equivalence margin was prespecified and the study was designed to evaluate between-group differences rather than equivalence or noninferiority.

Consistent with the fatigue findings, perceived energy improved substantially after birth in both groups, without evidence of differential improvement. This differs from the trial by Vaziri et al., in which spontaneous pushing in the lateral position was associated with lower fatigue, and from the pooled analysis by Kownaklai et al., which favored spontaneous pushing for fatigue at approximately 2 h postpartum [7,10]. Differences in assessment timing and intervention composition may partly explain these findings. A transient reduction in exertional fatigue soon after birth may no longer be apparent at 24 h. In addition, the Vaziri intervention combined pushing technique with lateral positioning, preventing separation of their individual effects [7].

Beyond maternal recovery, physiological pushing was associated with a lower likelihood of longer second-stage duration. A greater proportion of women in this group were in the shortest duration category and a smaller proportion were in the longest; the adjusted proportional-odds analysis yielded an OR of 0.20 (95% CI, 0.09 to 0.45; *p* < 0.001). The group-specific proportional-odds test showed no evidence of assumption violation, and the association remained significant after Holm correction. The direction of this association is consistent with findings by Yildirim and Beji, Lee et al., and Araújo et al., who reported favorable duration or active-pushing outcomes with spontaneous or undirected techniques [6,8,15]. By contrast, other studies reported shorter labor with coached or Valsalva pushing [9,16,17]. Cross-study comparisons may be complicated by differences in what is timed. Physiological management can include passive descent before the urge to push, potentially lengthening total second-stage duration even when active expulsive effort is shorter. Open- versus closed-glottis coaching has been evaluated as a distinct component of directed pushing [18]. Maternal position can influence second-stage duration [19], while epidural analgesia may also alter second-stage management [20]. Differences in pushing technique and related birth outcomes add further heterogeneity [21]. In the present study, sequential-period allocation and unmeasured clinical and provider-related differences may have influenced the observed association. It is therefore best interpreted as exploratory rather than causal. Because the available second-stage measure captured the interval from complete cervical dilatation to birth, the timing of active-pushing onset was not available as a separate measure. Accordingly, passive descent and active expulsive effort could not be evaluated independently within the recorded second-stage duration.

In contrast to the second-stage-duration association, no consistent between-group pattern was observed for maternal clinical outcomes. Spontaneous vaginal delivery, blood loss ≤500 mL, and intact perineum did not differ after multiplicity correction, and their numerical directions did not uniformly favor either approach. This pattern is consistent with the Cochrane review, which found no clear difference in spontaneous vaginal birth, severe perineal laceration, or episiotomy [3], and with Yao et al., who found no difference in spontaneous vaginal birth despite lower Caesarean-section and extended-episiotomy rates in some pooled comparisons [4]. Findings from individual studies are likewise mixed [15,22], and broader evidence suggests that perineal outcomes may reflect pushing technique and other aspects of second-stage perineal management [21,23]. Blood loss is additionally influenced by uterine tone, placental factors, genital-tract trauma, augmentation, and labor duration [24]. In this study, blood loss was clinically estimated and categorized rather than objectively quantified, allowing potential measurement error and threshold misclassification. Although biomechanical modeling suggests that repetitive loading may contribute to pelvic-floor tissue fatigue [25], mechanistic evidence does not establish universal clinical protection from either pushing technique. Overall, the recorded maternal outcomes did not show a consistent between-group pattern, while uncertainty remains for less frequent events.

Variation in intrapartum management provides additional context for these findings. Oxytocin augmentation and supplemental oxygen administration were recorded as co-interventions and were not components of either pushing protocol. Oxytocin was administered to 2 of 60 women (3.3%) in the physiological-pushing group and 7 of 60 (11.7%) in the directed-pushing group; the corresponding frequencies of oxygen administration were 5 of 60 (8.3%) and 8 of 60 (13.3%), respectively. Oxytocin use was retained as a covariate in the revised adjusted models. Oxygen administration was infrequent, and detailed information regarding its indication, timing, duration, and dose was unavailable, limiting more specific evaluation of its role. The observed differences in these co-interventions may reflect variation in intrapartum management rather than effects of pushing technique. Residual confounding from other management factors remains possible because cervical status and induction procedures may influence obstetric outcomes [26], while epidural analgesia may also affect second-stage management and outcomes [20].

A similarly neutral pattern was observed for neonatal outcomes. Good neonatal condition at 1 min was slightly more frequent with physiological pushing, the 5 min outcome was identical, and absence of neonatal intensive care unit admission numerically favored directed pushing; none of these outcomes differed after multiplicity correction. This overall pattern is consistent with three reviews that found no reproducible advantage for major neonatal or Apgar outcomes [3,4,10]. It differs from Yildirim and Beji, who reported higher Apgar scores and more favorable cord-gas measures with spontaneous pushing, and from Lee et al., who reported more adverse neonatal outcomes with directed pushing among women without regional analgesia [6,8]. Differences in outcome sensitivity and clinical practice may partly account for these findings. Apgar scores provide a clinical assessment of neonatal adaptation after birth but do not directly measure acid–base status, while neonatal intensive care unit admission may also be influenced by local clinical practice. Importantly, an abnormal early Apgar score does not necessarily indicate a corresponding disturbance in umbilical-cord acid–base status; for example, a retrospective cohort reported differences in 1 min Apgar status without a corresponding difference in neonatal arterial blood-gas findings [27]. In the present cohort, umbilical-cord blood gases, including cord-blood pH and base excess, were not measured; therefore, neonatal acidemia could not be evaluated directly. The neonatal findings should consequently be interpreted as showing no difference detected in the recorded clinical outcomes rather than as evidence of neonatal equivalence or absence of acid–base disturbance.

### 4.1. Strengths and Limitations

Strengths of this study include complete follow-up for fatigue and perceived energy, adjustment for the corresponding baseline scores and measured confounders, participant-level mixed-effects modeling, Holm adjustment for the prespecified family of secondary outcomes, and an overlap-weighted sensitivity analysis. These complementary approaches produced similar estimates for the primary outcome.

Despite these strengths, several limitations should be acknowledged. First, some limitations were inherent to the non-randomized, sequential-period study design. Allocation according to the implementation period introduced the potential for selection bias, baseline imbalance, and temporal changes. Although adjusted models and propensity-score weighting were applied, these approaches account only for recorded covariates. Residual confounding therefore cannot be ruled out and is an inherent consideration when interpreting this type of non-randomized study.

In addition, some variables and outcomes were not separately collected or objectively measured. The same data-collection framework was applied to both groups; therefore, the absence of these data represented a study-wide limitation rather than selective reporting in either group. Gravidity was recorded and incorporated into the analyses, whereas parity, regional or epidural analgesia, detailed maternal position, provider identifiers, and real-time protocol adherence were unavailable. Gravidity was not considered a surrogate for parity because multigravidity does not necessarily indicate a previous birth. Consequently, potential group differences in these factors, as well as provider-related effects, protocol deviations, crossover, or contamination, were not directly assessed. Maternal blood loss was estimated rather than objectively quantified in both groups, and cord blood-gas data were unavailable for both groups. Thus, data availability was comparable between the groups, although the distributions of these unmeasured factors remain unknown. Documentation of staff training and individual-level intervention fidelity was not available for analysis. In addition, active-pushing onset was not recorded separately, so active-pushing duration could not be distinguished from the overall second-stage duration.

Finally, the measurement characteristics of the Arabic-translated VAS-F should be considered. Fatigue-domain Cronbach’s α values ranged from 0.504 to 0.617, suggesting modest internal consistency, whereas perceived-energy coefficients ranged from 0.735 to 0.772. Measurement imprecision in the fatigue domain may have increased variability and reduced sensitivity to small between-group differences. Because the same instrument was administered to both groups, this imprecision would be expected to affect both groups similarly rather than favor either pushing technique. Although the original VAS-F was validated in other populations [13], formal cross-cultural validation among Saudi or Arabic-speaking postpartum women has not been established, and the archived documentation did not provide complete details of every translation stage. These measurement considerations may therefore have reduced sensitivity to small differences, while the common use of the instrument in both groups limits concern about systematic favoring of either technique.

### 4.2. Recommendations for Future Research

Building on these findings, future research can examine physiological and directed pushing across diverse maternity settings and over a broader postpartum period. Prospective multicenter investigations could clarify whether the observed recovery patterns remain consistent across different care environments and whether any effects are immediate or sustained. Formal cross-cultural validation of the Arabic VAS-F in postpartum populations would further strengthen the interpretation of fatigue and perceived-energy outcomes. Integrating maternal-reported recovery with standardized obstetric and neonatal outcomes may ultimately support individualized, woman-centered approaches to second-stage care [10,28].

## 5. Conclusions

At 24 h postpartum, no between-group differences were detected in maternal fatigue or perceived energy, and no consistent pattern favoring either approach was observed across the other evaluated maternal and neonatal outcomes. Physiological pushing was associated with a lower likelihood of longer second-stage duration, although this secondary finding should be interpreted cautiously given the non-randomized design. The findings support individualized pushing care based on maternal preferences and clinical circumstances. Future multicenter randomized research using culturally validated recovery measures may further clarify the comparative effects of these approaches.

## Figures and Tables

**Table 1:** Participant and Intrapartum Characteristics by Pushing Group (N = 120).

Characteristic	Category	Physiological Pushing (*n* = 60)	Directed Pushing (*n* = 60)	SMD
**Age, years (mean [SD])**	–	25.97 (4.35)	27.62 (4.88)	0.357
**Body mass index, kg/m^2^ (mean [SD])**	–	27.65 (4.81)	30.03 (4.93)	0.488
**Education (n [%])**	Less than secondary	11 (18.3)	16 (26.7)	0.438
	Secondary	17 (28.3)	24 (40.0)	
	Bachelor	31 (51.7)	20 (33.3)	
	Postgraduate	1 (1.7)	0 (0.0)	
**Residence (n [%])**	Makkah	59 (98.3)	53 (88.3)	0.409
	Other	1 (1.7)	7 (11.7)	
**Occupation (n [%])**	Housewife	53 (88.3)	50 (83.3)	0.266
	Student	6 (10.0)	10 (16.7)	
	Working	1 (1.7)	0 (0.0)	
**Gravidity (n [%])**	Primigravida	33 (55.0)	23 (38.3)	0.339
	Multigravida	27 (45.0)	37 (61.7)	
**Exercise during pregnancy (n [%])**	No	41 (68.3)	40 (66.7)	0.036
	Yes	19 (31.7)	20 (33.3)	
**Kegel exercise (n [%])**	No	56 (93.3)	53 (88.3)	0.174
	Yes	4 (6.7)	7 (11.7)	
**Smoking (n [%])**	No	57 (95.0)	58 (96.7)	0.083
	Yes	3 (5.0)	2 (3.3)	
**Gestational age (n [%])**	≥37–<38 weeks	7 (11.7)	6 (10.0)	0.139
	≥38–<40 weeks	36 (60.0)	40 (66.7)	
	≥40 weeks	17 (28.3)	14 (23.3)	
**First-stage duration (n [%])**	<10 h	17 (28.3)	23 (38.3)	0.251
	≥10–<12 h	9 (15.0)	7 (11.7)	
	≥12–<19 h	21 (35.0)	16 (26.7)	
	≥19 h	13 (21.7)	14 (23.3)	
**Membrane condition (n [%])**	Intact	0 (0.0)	2 (3.3)	0.332
	SROM	28 (46.7)	30 (50.0)	
	ARM	31 (51.7)	28 (46.7)	
	PROM	1 (1.7)	0 (0.0)	
**Oxytocin augmentation (n [%])**	No	58 (96.7)	53 (88.3)	0.320
	Yes	2 (3.3)	7 (11.7)	
**Oxygen use (n [%])**	No	55 (91.7)	52 (86.7)	0.161
	Yes	5 (8.3)	8 (13.3)	

**Note.** Continuous variables are presented as mean (SD), and categorical variables as n (%). SMDs are absolute standardized mean differences. Values < 0.10, 0.10 to <0.20, 0.20 to <0.50, and ≥0.50 indicate negligible, small, moderate, and large imbalance, respectively. Significance tests were not performed because SMDs, rather than *p* values, were prespecified for balance assessment. ARM, artificial rupture of membranes; PROM, prelabor rupture of membranes; SD, standard deviation; SMD, standardized mean difference; SROM, spontaneous rupture of membranes.

**Table 2:** Unadjusted Fatigue and Perceived-Energy Scores Across Assessments.

Domain	Assessment	Physiological, Mean (SD)	Directed, Mean (SD)	Cronbach’s Alpha
**Fatigue**	End of first stage	46.40 (3.86)	47.13 (4.45)	0.504
	1 h postpartum	39.17 (5.47)	37.63 (5.49)	0.617
	24 h postpartum	31.25 (5.50)	30.92 (5.02)	0.594
**Perceived energy**	End of first stage	9.43 (2.24)	9.60 (2.60)	0.735
	1 h postpartum	13.60 (3.18)	13.90 (3.78)	0.746
	24 h postpartum	17.88 (3.30)	18.52 (3.02)	0.772

**Note.** Values are mean (SD). Higher domain scores indicate greater fatigue or greater perceived energy, respectively. Cronbach’s alpha summarizes internal consistency at each assessment. SD, standard deviation; VAS-F, Visual Analogue Scale for Fatigue.

**Table 3:** Adjusted Primary, Longitudinal, and Sensitivity Analyses.

Domain	Analysis or Model Term	Estimate (95% CI) or Test Statistic	*p* Value
**Fatigue**	Adjusted 24 h difference (physiological minus directed)	0.20 (−2.05 to 2.45)	0.860
	Mixed-effects model: group	F(1, 107) = 0.05	0.817
	Mixed-effects model: time	F(2, 236) = 319.38	<0.001
	Mixed-effects model: group × time	F(2, 236) = 1.67	0.191
	Overlap-weighted sensitivity analysis	0.25 (−1.79 to 2.29)	0.809
**Perceived energy**	Adjusted 24 h difference (physiological minus directed)	−0.79 (−2.18 to 0.59)	0.260
	Mixed-effects model: group	F(1, 107) = 0.39	0.534
	Mixed-effects model: time	F(2, 236) = 315.52	<0.001
	Mixed-effects model: group × time	F(2, 236) = 0.24	0.785

**Note.** Adjusted differences are physiological minus directed. The 24 h fatigue model adjusted for baseline fatigue, age, body mass index, education, gravidity, gestational age, first-stage duration, and oxytocin use; the perceived-energy model used the corresponding baseline energy score. Mixed-effects models included a participant-specific random intercept, group, time, the group × time interaction, and the revised covariate set. The propensity-score model for the overlap-weighted analysis included age, body mass index, education, gravidity, gestational age, first-stage duration, oxytocin use, and baseline fatigue. CI, confidence interval.

**Table 4:** Comparison of Favorable Maternal and Neonatal Outcomes Between the Physiological- and Directed-Pushing Groups.

Outcome	Physiological, n (%)	Directed, n (%)	Exact Odds Ratio (95% CI)	Exact *p* Value	Holm-Adjusted *p* Value
Spontaneous vaginal delivery	55 (91.7)	60 (100.0)	0.00 (0.00–1.06)	0.057	0.401
Blood loss ≤ 500 mL	56 (93.3)	59 (98.3)	0.24 (0.00–2.52)	0.364	1.000
Intact perineum	9 (15.0)	17 (28.3)	0.45 (0.16–1.19)	0.120	0.719
Good neonatal condition at 1 min	46 (76.7)	44 (73.3)	1.19 (0.48–2.99)	0.833	1.000
Good neonatal condition at 5 min	58 (96.7)	58 (96.7)	1.00 (0.07–14.22)	1.000	1.000
No NICU admission	56 (93.3)	59 (98.3)	0.24 (0.00–2.52)	0.364	1.000

**Note.** Exact odds ratios compare physiological with directed pushing for the favorable outcome shown. Holm-adjusted *p* values correspond to the prespecified family of six secondary outcomes. CI, confidence interval; NICU, neonatal intensive care unit.

## Data Availability

The data presented in this study are available on request from the corresponding author due to ethical and privacy restrictions related to the confidentiality of individual-level maternal and neonatal participant data.

## Supplementary Materials

The following supporting information can be downloaded at https://www.mdpi.com/article/10.3390/healthcare14193319/s1: Table S1. Covariate Balance Before and After Propensity-Score Overlap Weighting. Figure S1. Propensity-Score Distribution and Overlap by Pushing Group.

## Abbreviations

ARM, artificial rupture of membranes; BMI, body mass index; CI, confidence interval; HC3, heteroskedasticity-consistent covariance estimator type 3; LMM, linear mixed-effects model; NICU, neonatal intensive care unit; OR, odds ratio; PROM, prelabor rupture of membranes; SD, standard deviation; SMD, standardized mean difference; SROM, spontaneous rupture of membranes; TIDieR, Template for Intervention Description and Replication; TREND, Transparent Reporting of Evaluations with Nonrandomized Designs; VAS-F, Visual Analogue Scale for Fatigue.
