# Supplementary material for: Physiological Versus Directed Pushing During the Second Stage of Labor: A Non-Randomized Quasi-Experimental Study of Maternal Recovery and Clinical Outcomes
Source: Healthcare (Basel). 2026 Oct 6;14(19):3319. doi: 10.3390/healthcare14193319 (PMC13665035; doi:10.3390/healthcare14193319)
Supplement: Supplementary file 1 [file healthcare-14-03319-s001.zip › healthcare-4574415-supplementary.pdf]

Supplementary Material

Table S1. Covariate Balance Before and After Propensity-Score Overlap Weighting

**Covariate**	**Absolute SMD before weighting**	**Absolute SMD after weighting**
Age, years	0.36	<0.001
BMI, kg/m²	0.493	<0.001
Education: Secondary	0.248	<0.001
Education: Bachelor/Postgraduate	0.412	<0.001
Gravidity: Multigravida	0.339	<0.001
Gestational age: ≥38–<40 weeks	0.139	<0.001
Gestational age: ≥40 weeks	0.114	<0.001
First-stage duration: ≥10–<12 h	0.098	<0.001
First-stage duration: ≥12–<19 h	0.181	<0.001
First-stage duration: ≥19 h	0.04	<0.001
Oxytocin augmentation: Yes	0.32	<0.001
Baseline fatigue score	0.177	<0.001

Note. SMD, standardized mean difference. Propensity scores were estimated using age, body mass index, education, gravidity, gestational age, first-stage duration, oxytocin augmentation, and baseline fatigue. All post-weighting absolute SMDs were <0.001.

Figure S1. Propensity-Score Distribution and Overlap by Pushing Group

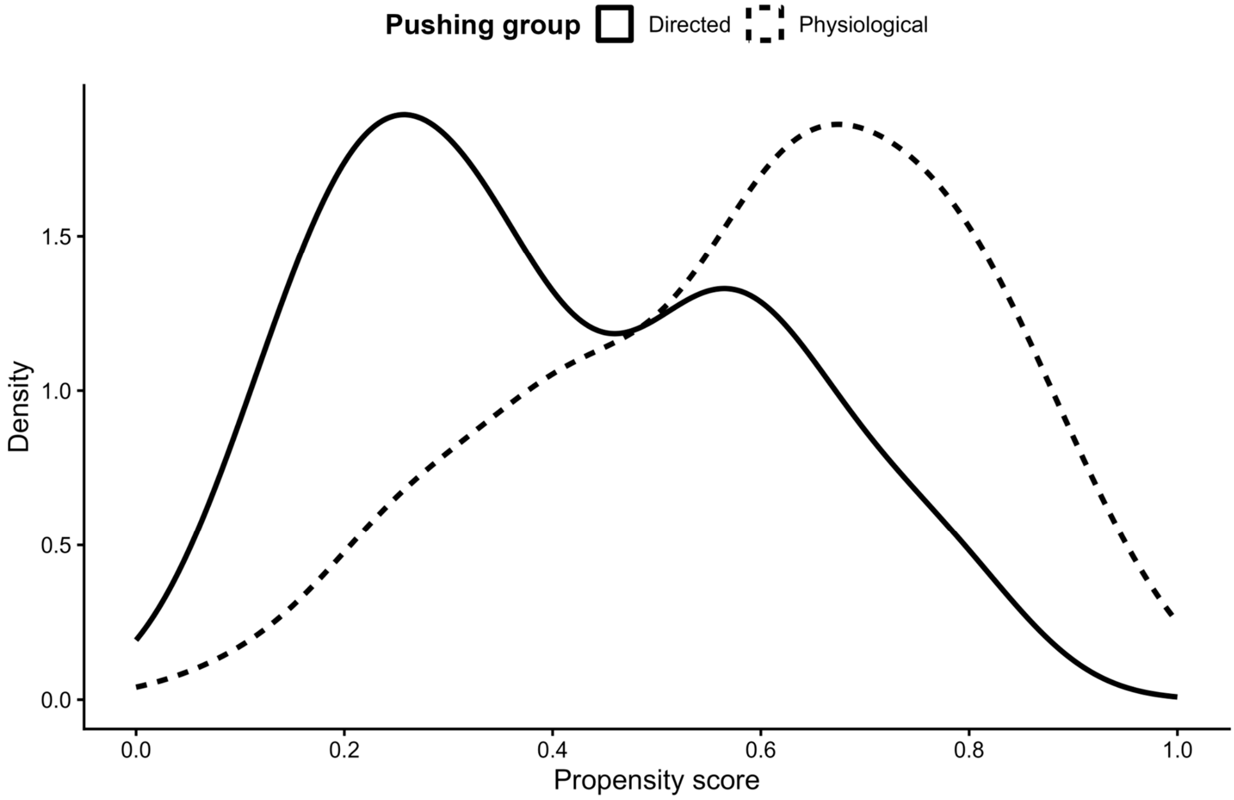

Figure S1. Distribution of estimated propensity scores in the physiological- and directed-pushing groups before overlap weighting. The figure displays the degree of common support used for the overlap-weighted sensitivity analysis.
